# Correlation between serum IGF-1 and EGF levels and neuropsychiatric and cognitive in Parkinson’s disease patients

**DOI:** 10.1007/s10072-022-06490-1

**Published:** 2022-11-16

**Authors:** Xiaoxue Shi, Jinhua Zheng, Jianjun Ma, Dongsheng Li, Qi Gu, Siyuan Chen, Zhidong Wang, Wenhua Sun, Mingjian Li

**Affiliations:** 1 Department of Neurology, Henan Provincial People’s Hospital, Zhengzhou, China; 2 Department of Neurology, Zhengzhou University People’s Hospital, ZhengzhouHenan Province, 450003 China; 3 Department of Neurology, Henan University People’s Hospital, Zhengzhou, China

**Keywords:** IGF-1, EGF, Parkinson’s disease, Nonmotor symptoms

## Abstract

**Background:**

Insulin-like growth factor 1 (IGF-1) and epidermal growth factor (EGF) exert neuroprotective effects in Parkinson’s disease (PD). To date, studies on the relationships between serum IGF-1 and EGF levels and nonmotor symptoms in PD patients have been rare.

**Methods:**

A Siemens automatic chemical analyzer was used to determine serum IGF-1 levels, and enzyme-linked immunosorbent assay was used to detect serum EGF levels in 100 healthy controls and 100 PD patients, including those in the early (*n* = 49) and middle-late (*n* = 51) stage of the disease. Evaluation of motor symptoms and nonmotor symptoms in PD patients was assessed by the associated scales.

**Results:**

Serum IGF-1 and EGF levels were higher in PD patients than in healthy controls, and serum IGF-1 and EGF levels were higher in early stage PD patients than in middle-late stage PD patients. Serum IGF-1 levels were significantly negatively correlated with anxiety, depression, and cognitive dysfunction; serum EGF levels were significantly negatively correlated with cognitive dysfunction. Combining IGF-1 and EGF in the diagnosis of PD was more valuable than using a single factor in the diagnosis.

**Conclusions:**

This study shows that serum IGF-1 levels were correlated with the nonmotor symptoms of anxiety, depression, and cognitive dysfunction and that EGF levels were correlated with cognitive dysfunction. The combination of IGF-1 and EGF increased the value for a PD diagnosis. This is the first report of the simultaneous detection of IGF-1 and EGF levels to explore the correlation with nonmotor symptoms of PD.

## Introduction

Parkinson's disease (PD) is a chronic progressive neurodegenerative disorder characterized by the loss of substantia nigra dopaminergic neurons, with a prevalence of 1.7% in people 65 years of age and older, second only to Alzheimer’s disease[1, 2]. PD mainly manifests with motor symptoms, including bradykinesia, resting tremor, and muscle rigidity. In addition, nonmotor symptoms are often observed before and after motor symptoms and include cognitive impairment and neuropsychiatric symptoms[3]. At present, the diagnosis of PD mainly depends on the collection of medical history and clinical manifestations. Therefore, finding reliable markers for the diagnosis of PD and prediction of disease progression is a current research hotspot. The etiology and pathogenesis of PD remain unclear. Studies have found that neurotrophic factors (NTFs) decrease in nigrostriatal regions, which in turn reduces the concentration of dopamine (DA) in the brain, leading to the appearance of clinical symptoms of PD[4].

As a neurotrophic factor, insulin-like growth factor-1 (IGF-1) is a peptide hormone composed of 70 amino acids and is involved in nerve growth, differentiation, maturation, myelination, and survival[5, 6]. It has been shown that neurotoxicity from α-synuclein aggregation is mediated by DA, but even in the presence of DA, IGF exerts a protective effect against α-synuclein aggregation, which keeps developing neurons alive and protects mature neurons from excitotoxic damage[7, 8]. The substantia nigra (SN) contains a high density of IGF-1 receptors, and IGF increases the survival of brainstem neurons, including those in the SN, and rescues embryonic DA neurons from programmed cell death[9–11].

Epidermal growth factor (EGF) is a polypeptide growth factor with strong physiological activity that can regulate the growth, development, proliferation, and differentiation of neuronal cells. On the one hand, EGF can act as a powerful cell mitogen to accelerate the growth and development of the nervous system, and on the other hand, it can play the role of neurotrophic factor in nigrostriatal DA neurons[12, 13]. EGF upregulates tyrosine hydroxylase (TH) expression, increases DA turnover in the striatum, and inhibits dopaminergic neuron degeneration[14].

The relationship between neurotrophic factors and PD is currently unclear. Therefore, the purpose of our study was (1) to evaluate the levels of serum IGF-1 and EGF in PD patients and to further explore their predictive value regarding PD and (2) to investigate the relationship between neurotrophic factor levels and nonmotor symptoms in PD patients.

## Methods

### Patients

A total of 100 PD patients from the inpatient ward were consecutively recruited from 2020 to 2021. Patients were diagnosed by two experienced neurologists according to the UK PD Society Brain Bank Clinical Diagnostic Criteria for PD[15]. Patients with (1) atypical and secondary PD, (2) cardiovascular and cerebrovascular diseases, such as myocardial infarction and cerebral infarction, and (3) acute or chronic infections or surgical procedures within the previous 3 months were excluded. A total of 100 healthy volunteers participated in this study. All subjects signed written informed consent before participation.

### Clinical characteristics

General clinical data, such as sex and age, were recorded. Motor symptoms were evaluated by Part III of the Unified Parkinson’s Disease Rating Scale (UPDRS III)[16]. Nonmotor symptoms were evaluated by the Pittsburgh Sleep Quality Index (PSQI), Nonmotor Symptom Scale (NMSS), 14-item Hamilton Anxiety Rating Scale (HAMA-14), 17-item Hamilton Depression Rating Scale (HAMD-17), and Mini-Mental State Examination (MMSE). Hoehn and Yahr (H-Y) classification and the UPDRS were used to evaluate disease severity.

Hoehn Yahr (H-Y) staging was used to divide PD into 0 ~ 5 stages[17] (stage 1 ~ 2 is the early stage, and stage 2.5 ~ 5 is the middle and late stage). Individuals who scored ≥ 14 were considered to have anxiety[18]. Individuals who scored ≥ 8 were considered to have depression[19]. Individuals who scored ≤ 26 were considered to exhibit cognitive dysfunction[20].

For PD patients with symptom fluctuations, the evaluation of the movement symptoms was in the off period.

### Blood sampling

Between 07:30 and 08:30 am, fasting serum IGF-1 concentrations were determined by a Siemens automatic chemical analyzer (IMMULITE 2000-xpi), which operates on the principle of chemiluminescence, and serum EGF levels were detected by ELISA.

### Statistical analysis

Quantitative data with a normal distribution based on the *Kolmogorov–Smirnov test* are expressed as the means ± standard deviations, and *Student’s t tests* were used for comparisons between the two groups. Multiple groups of data consistent with a normal distribution and homogeneity of variance were compared by *one-way analysis of variance*, and post hoc *LSD t tests* were used to further compare differences in serum IGF-1 and EGF levels between the control group and the early-stage and middle/late-stage PD groups. Data that did not have a normal distribution are expressed as medians (quartile ranges), and the *Mann–Whitney U test* was used for comparisons. The identification of PD patients with IGF-1 and EGF levels and their combination was evaluated by receiver operating characteristic (ROC) curve analysis. *Spearman’s correlation analysis* was used to evaluate correlations between the serum IGF-1 levels and various indicators. All tests were two-tailed, and a *probability (P) value* of less than 0.05 was considered statistically significant. The Statistical Package for the Social Sciences (SPSS) program version 26.0 was used for all statistical analyses.

## Results

### Demographic data and serum IGF-1 and EGF levels in healthy controls and PD patients

No differences were found between the PD and control groups in age (63.37 ± 8.99 vs. 62.96 ± 6.37, *t* = 0.372, *P* = 0.710) or sex (56% vs. 52%, *c2* = 0.322, *P* = 0.570), while serum IGF-1 and EGF levels were significantly higher in the PD patients than in the healthy controls (IGF-1: 149.50 ± 33.85 mmol/L vs. 99.06 ± 21.29 mmol/L, P < 0.001; EGF: 62.96 ± 11.72 pg/mL vs. 52.22 ± 9.24 pg/mL, *P* < 0.001) (Table 1).

**Table 1:** Demographic data and serum levels of IGF-1 and EGF in the PD group and healthy control group

	PD(*n* = 100)	HC(*n* = 100)	*t/χ*^2^	*P*
Sex(male/female)	56/44	52/48	0.322	0.570
Age(yr)	63.37 ± 8.99	62.96 ± 6.37	0.372	0.710
IGF-1(mmol/L)	149.50 ± 33.85	99.06 ± 21.29	− 12.61	< 0.001
EGF(pg/mL)	62.96 ± 11.72	52.22 ± 9.24	− 7.20	< 0.001

**Fig. 1:**
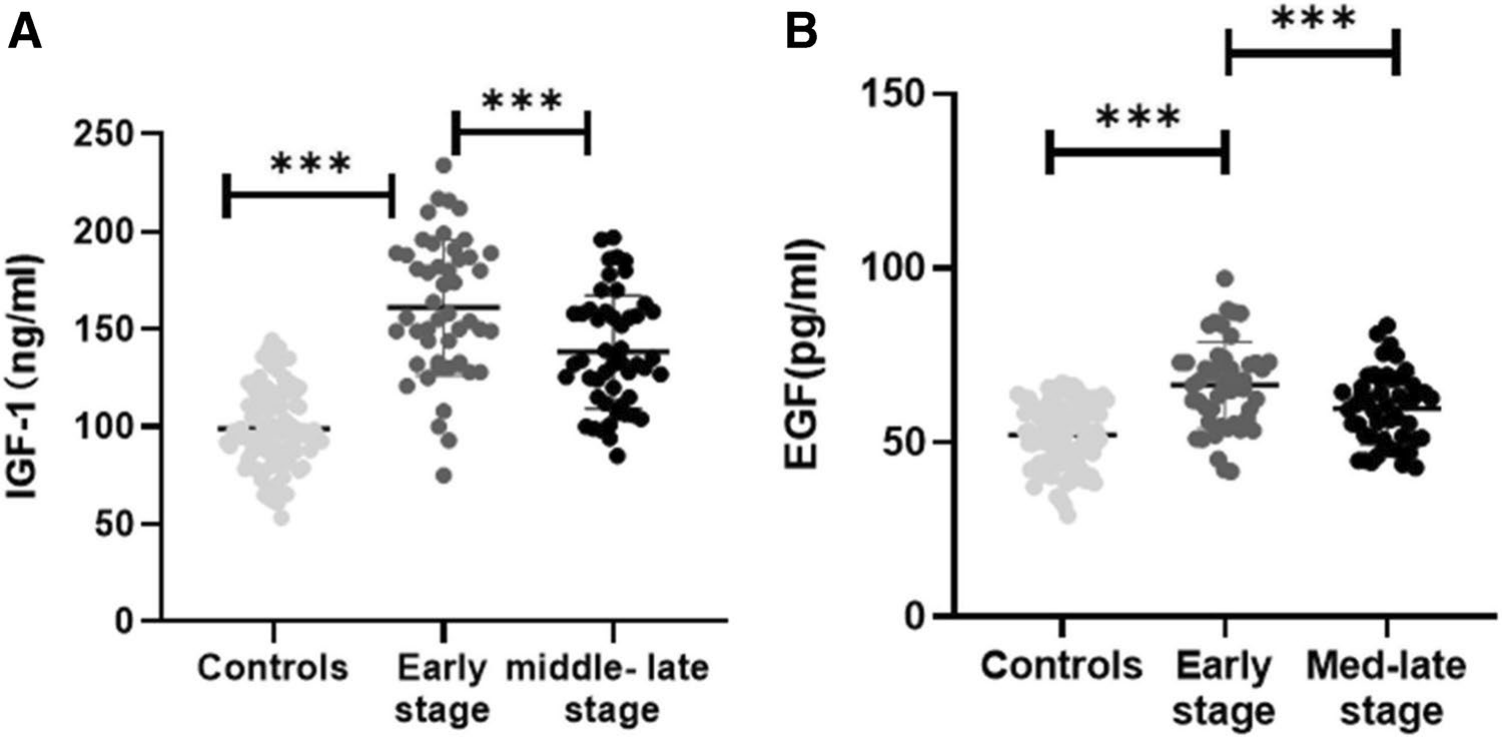
Distribution of serum IGF-1 and EGF levels in PD patients classified by Hoehn-Yahr stages. (**A**) Distribution of serum IGF-1 levels in different H-Y stages. (**B**) Distribution of serum EGF levels in different H-Y stages

Based on the H-Y classification, the PD patients were divided into early-stage (*n* = 49) and middle-late stage PD patients (*n* = 51). IGF-1 and EGF levels in the early stage and middle-late stage PD groups were higher than those in the healthy control group, and the differences between the three groups were statistically significant (IGF-1: 2 = 94.89, *df* = 2, *P* < 0.001; EGF: 2 = 32.60, *df* = 2, *P* < 0.001) (Fig. 1A, B).

### ROC curve analysis of IGF-1 and EGF levels and their combination in the diagnosis of PD

**Fig. 2:**
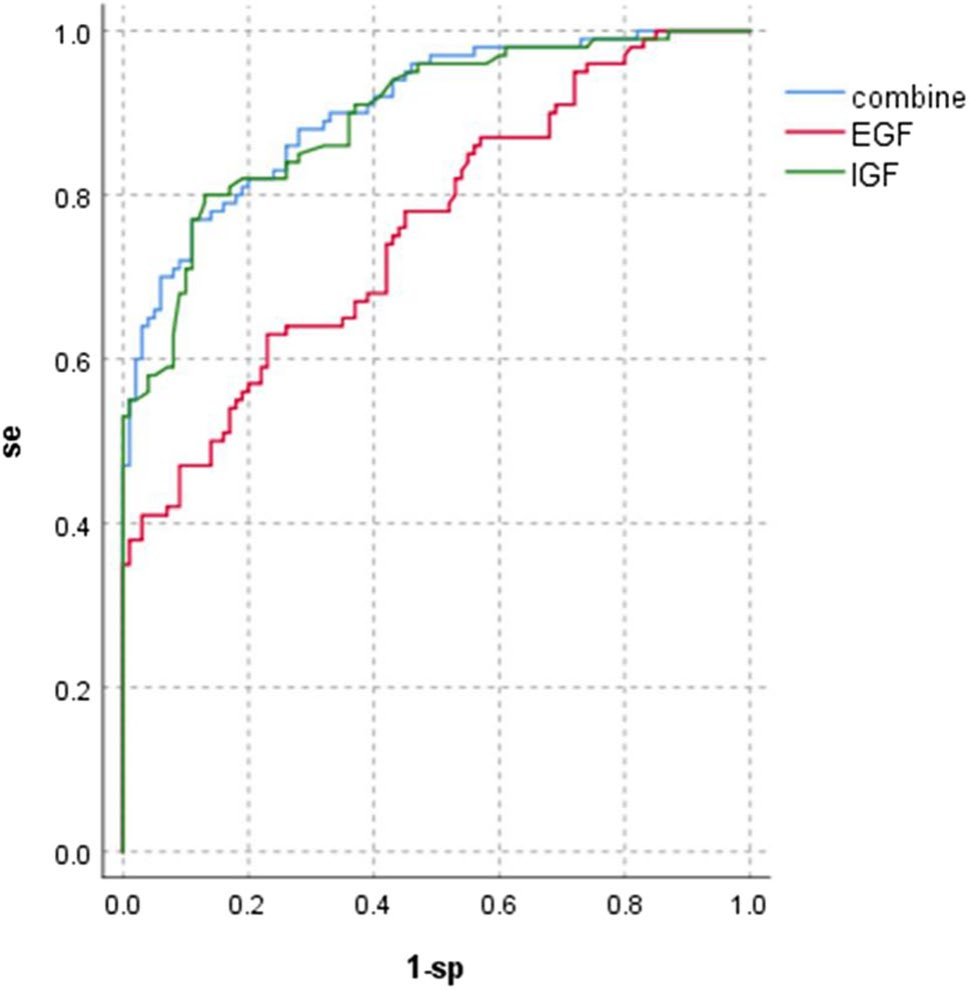
ROC curves for IGF-1, EGF, and their combination in the diagnosis of PD

Serum IGF-1 and EGF levels differentiated PD and healthy controls. Based on an ROC curve analysis, the identification of PD patients with serum IGF-1 levels had an area under the curve (AUC) value of 0.895, sensitivity of 87%, specificity of 80%, and cutoff value of 123.8 mmol/L, and the identification of PD patients with serum EGF levels had an area under the curve (AUC) value of 0.758, sensitivity of 77%, specificity of 63%, and cutoff value of 59.45 pg/mL. After we combined the two measures for the diagnosis of PD, we concluded that the identification of PD patients with both serum EGF and EGF levels had an area under the curve (AUC) value of 0.904, sensitivity of 89%, and specificity of 77% (Table 2; Fig. 2).

**Table 2:** ROC analysis of IGF-1, EGF, and their combination in the diagnosis of PD

	AUC	SE	95%	*P*	Cutoff value	se	sp
IGF-1	0.895	0.021	0.852-0.938	<0.001	123.8	87%	80%
EGF	0.758	0.033	0.693-0.823	<0.001	59.45	77%	63%
combine	0.904	0.020	0.864-0.944	<0.001	-	89%	77%

### Correlation of IGF-1 and EGF with disease severity in PD patients

**Fig. 3:**
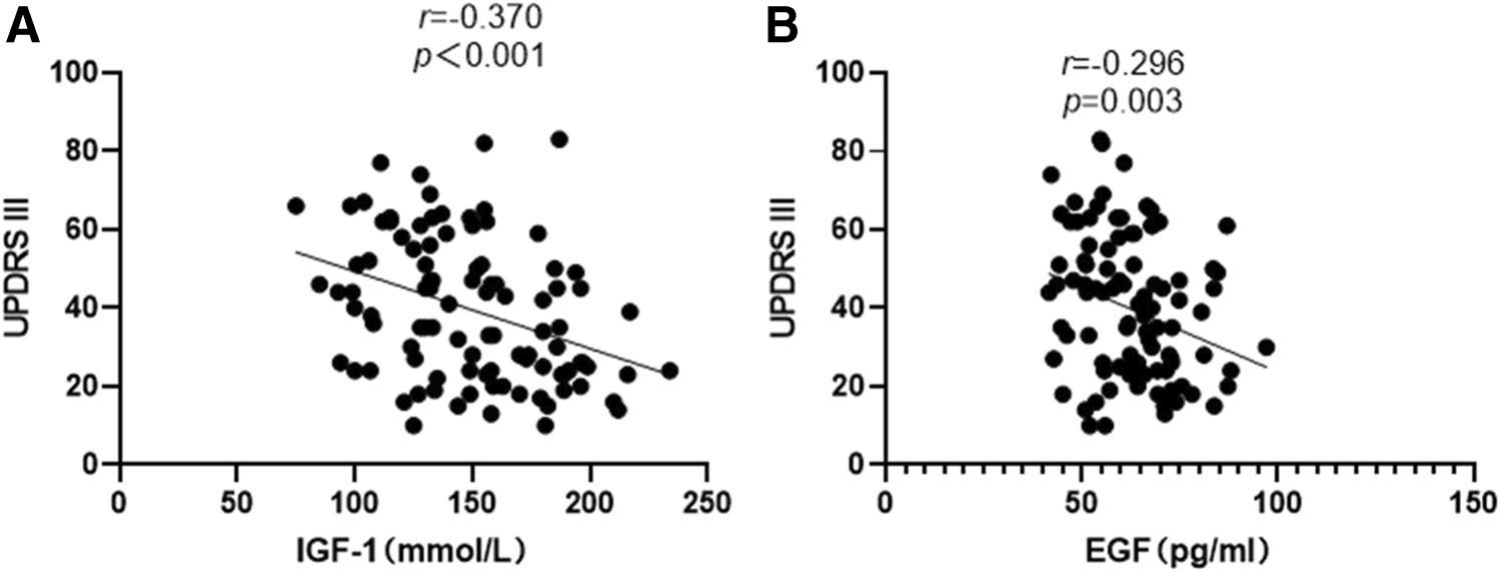
Correlations between serum IGF-1 and EGF levels and disease duration and disease severity in PD patients. (**A**)Serum IGF-1 levels in PD patients were negatively correlated with UPDRS III scores (*Pearson rank* =  − 0.370, *P* < 0.001, *n* = 100). (**B**) Serum EGF levels in PD patients were negatively correlated with UPDRS III scores (*Pearson rank* =  − 0.296, *P* = 0.003, *n* = 100)

We found a negative correlation between serum IGF-1 levels and UPDRS III scores in PD patients (*r* =  − 0.370, *P* < 0.001) and a negative correlation between serum EGF levels and UPDRS III scores in PD patients (*r* =  − 0.296, *P* = 0.003) (Fig. 3).

### Correlation analysis of nonmotor symptoms with serum IGF-1 and EGF levels in PD patients

Correlation analysis showed that IGF-1 levels were negatively correlated with NMSS total scores (*r* =  − 0.430, *P* < 0.001), HAMA-14 total scores (*r* =  − 0.311, *P* = 0.002), and HAMD-24 total scores (*r* =  − 0.331, *P* = 0.001) and positively correlated with MMSE total scores (*r* = 0.366, *P* < 0.001). EGF levels were negatively correlated with NMSS total scores (*r* =  − 0.314, *P* = 0.001) and positively correlated with MMSE total scores (*r* = 0.325, *P* = 0.001). The remaining nonmotor symptoms were not related to serum IGF-1 and EGF levels (*P* > 0.05) (Table 3).

**Table 3:** Correlation analysis of nonmotor symptoms and serum IGF-1 and EGF levels in PD patients

	Medians (quartile ranges)/means (standard deviations)	IGF-1		EGF	
		*Spearman*	*P*	*Spearman*	*P*
NMSS score	41 (21,72)	− 0.430*	< 0.001	− 0.314*	0.001
PSQI score	9 (5,13)	− 0.07	0.49	0.031	0.762
HAMA-14 score	10 (6,15)	− 0.311^*^	0.002	− 0.177	0.079
HAMD-24 score	11 (7,16)	− 0.331^*^	0.001	− 0.069	0.498
MMSE score	27 (24,29)	0.366*	< 0.001	0.325*	0.001

**Fig. 4:**
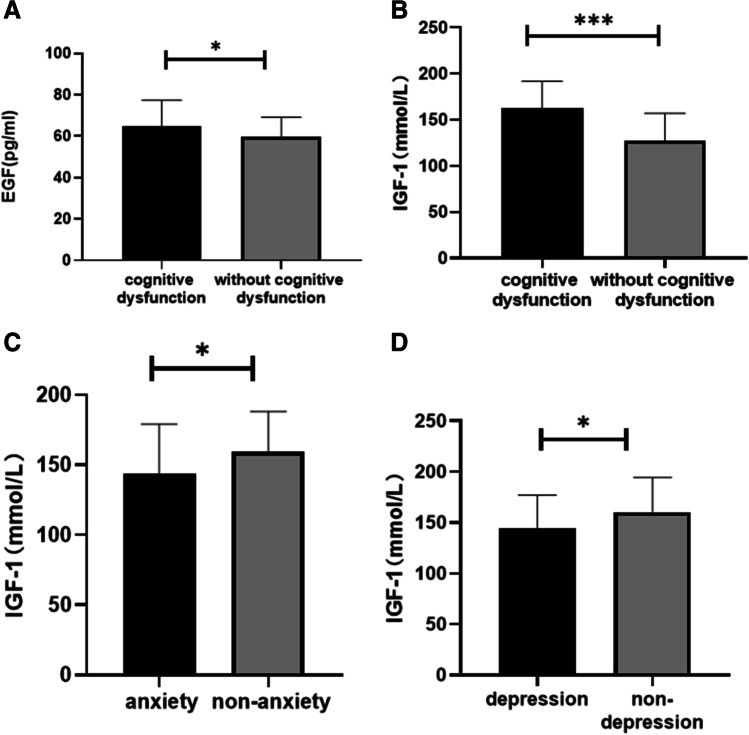
(**A**) Serum EGF levels in subgroups of PD patients with or without cognitive impairment. (**B**) Serum IGF-1 levels in subgroups of PD patients with or without cognitive impairment. (**C**) Serum IGF-1 levels in subgroups of PD patients with or without anxiety. (**D**) Serum IGF-1 levels in subgroups of PD patients with or without depression

Next, we used the significantly correlated nonmotor symptoms as grouping criteria for the PD patients to compare differences in serum IGF-1 and EGF concentrations between each pair of subgroups. Among the PD patients, serum IGF-1 levels were lower in the anxiety subgroup, depression subgroup, and cognitive dysfunction subgroup than in the subgroups without anxiety (143.66 ± 35.47 mmol/L vs*.* 159.46 ± 28.70 mmol/L, *P* = 0.023), depression (144.19 ± 32.78 mmol/L vs*.* 160.30 ± 33.99 mmol/L, *P* = 0.024), and cognitive dysfunction (136.36 ± 34.60 mmol/L vs. 160.70 ± 29.09 mmol/L, *P* < 0.001). Serum EGF levels in the PD patients were lower in the cognitive dysfunction subgroup than in the subgroup without cognitive dysfunction (58.92 ± 10.02 pg/mL vs. 66.41 ± 12.05 pg/mL, *P* = 0.001) (Fig. 4).

## Discussion

In our research, we concluded that serum IGF-1 levels in the PD patients were significantly higher than those in healthy controls, and with the progression of the disease, IGF-1 levels gradually decreased. To further explore the relationships between serum IGF-1 levels and PD disease progression and severity, we analyzed the differences in IGF-1 levels in patients in different H-Y stages, and the results showed that serum IGF-1 levels in early-stage PD were significantly higher than those in the middle/late stages. In addition, this study concluded that serum IGF-1 levels were negatively correlated with UPDRS III scores. DA can trigger apoptosis, an active program of cellular self-destruction, in various neuronal cultures, so inappropriate activation of apoptosis by DA and its oxidative products may cause cell death in the substantia nigra in PD[21]. IGF-1 can protect neuronal cells in a harmful microenvironment and has a protective effect on DA-induced neurotoxicity[22]. As a neuroprotective factor, IGF-1 may be elevated in a compensatory manner in early PD to protect dopaminergic neurons from degeneration[23].

The levels of IGF-1 gradually decrease in the middle/late stages of PD, and we speculate that the possible reasons are as follows: on the one hand, the activity of the growth hormone GH-IGF-1 axis gradually decreases with increasing age, which may promote the development of neurodegenerative diseases such as PD in the aging process; on the other hand, during the course of the disease, due to the depletion of the neuroprotective effects of IGF-1, the levels of IGF-1 decrease in the middle/late stages[22].

Our analysis showed that serum EGF levels in the PD patients were significantly higher than those in the healthy controls, and with the progression of the disease, EGF levels gradually decreased and serum EGF levels in the PD patients were negatively correlated with UPDRS III scores. Currently, the pathological mechanisms leading to elevated EGF levels in PD are currently unclear. The EGF upregulates the expression of tyrosine hydroxylase (TH) in rats, improves striatal DA turnover, and inhibits DA neuron degeneration[14]. Although EGF administration rescued TH immunoreactivity in the peristriatal region, it did not prevent the overall neurodegeneration of SN dopaminergic cells[13].

Phosphatidylinositol 3-kinase (PI3-kinase) has been shown to be involved in synaptic plasticity by modulating neurotransmitter release and activating and enhancing neurotransmitter release in the hippocampus and nigrostriatal regions. It plays an important role in cell growth, proliferation, and differentiation[24]. PI3-kinase is involved in the enhancement of neurotransmitter release through two distinct mechanisms: EGF can simultaneously activate mitogen-activated protein kinases (MAPKs) and PI3-kinases distributed in the plasma membrane, and IGF-1 activates PI3 kinase in the intracellular membrane; the two factors synergistically promote increased dopamine release[25].The ROC curve analysis in this study showed that the AUCs for IGF-1 and EGF in the diagnosis of PD were both high, and the AUC of the combination of the two substances in the diagnosis of PD was higher than the AUC of either factor alone. This finding indicates that the combination of IGF-1 and EGF has potential value as a PD biomarker, providing a new approach to clinical PD diagnosis.

Higher levels of α-synuclein and diffuse β-amyloid have been observed in PD with dementia, both contributing to the development and progression of cognitive impairment[26, 27]. On the one hand, IGF-1 can increase the clearance of β-amyloid and inhibit the toxicity of β-amyloid; on the other hand, IGF-1 inhibits α-synuclein toxicity and aggregation by activating the cellular Akt pathway. The relationship between serum EGF and cognition in PD patients was analyzed, and it was concluded that serum EGF levels were negatively correlated with the occurrence of cognitive impairment. Low EGF levels are associated with frontal and temporal lobe-mediated changes in cognitive function and predict cognitive decline. EGF can increase the proliferation of neurons and glial cells in the hippocampus and play a neurotrophic role in hippocampal neurons. As the levels of EGF decrease, its neurotrophic function gradually declines followed by a gradual decline in cognitive function[28].

Neuropsychiatric symptoms such as anxiety and depression are very common among PD patients and are closely related to the decline in patient quality of life[29]. In this study, serum IGF-1 levels were negatively correlated with HAMD and HAMA scores. IGF-1 promotes hippocampal neurogenesis and can improve anxiety-like behaviors[30]. In the forced swim test with rats, IGF-1 exerts neurotrophic effects to produce antidepressant-like effects[31]. The metabolite of IGF-1, cyclic glycine-proline (cGP), can exert neuroprotective effects by improving the function of IGF-1. After supplementation with blackcurrant anthocyanins (BCA), cGP content increased, and anxiety and depression scores in PD patients decreased, presumably related to the neurotrophic function of IGF-1[32]. After 2 weeks of antidepressant treatment with serotonin (5-HT) in rats, IGF-1 concentrations in the hippocampus were significantly increased compared with controls[33]. Although 5-HT and IGF-1 receptors are structurally and biologically distinct, the downstream signaling pathways of IGF-1 and 5-HT exhibit a high degree of overlap, and both play a role in signal transduction pathways. Their synergistic effects were shown to enhance neuronal synaptic plasticity[34].

In our study, we concluded that serum IGF-1 and EGF levels in patients with Parkinson’s disease were associated with neuropsychiatric and cognitive function, but we admit that these associations, as well as those of IGF-1 and EGF with nonmotor symptoms, need to be further validated for the following main reasons. First, this is a cross-sectional study, and the nonmotor symptom scale assessment and the factor levels were measured only at specific time points; dynamic monitoring was not performed. Second, the participants in this study were from the same clinical center. Therefore, the next step is to conduct clinical multicenter studies with more subjects and conduct dynamic monitoring of the levels of these factors.

## Conclusions

This study shows that serum IGF-1 levels were correlated with the nonmotor symptoms of anxiety, depression, and cognitive dysfunction and that EGF levels were correlated with cognitive dysfunction. The combination of IGF-1 and EGF increased the value for a PD diagnosis. These findings indicate that monitoring IGF-1 and EGF may be potential biomarkers or treatment strategies for PD. Finally, to clarify the clinical significance of serum IGF-1 and EGF concentrations in PD patients, larger clinical and preclinical studies are needed to further explore the potential mechanism underlying changes in serum IGF-1 and EGF levels in patients with PD.
